# Per- and Polyfluoroalkyl Substances (PFAS) in Consumer Products: An Overview of the Occurrence, Migration, and Exposure Assessment

**DOI:** 10.3390/molecules30050994

**Published:** 2025-02-21

**Authors:** Yang Yang, Jin Wang, Shali Tang, Jia Qiu, Yan Luo, Chun Yang, Xiaojing Lai, Qian Wang, Hui Cao

**Affiliations:** 1 National Postdoctoral Research Station, Zhejiang Institute of Quality Sciences, Hangzhou 310018, China; tangshali_77@163.com (S.T.); qiuj@zjzay.com (J.Q.); luoy@zjzay.com (Y.L.); 2008f@163.com (C.Y.); laixj@zjzay.com (X.L.); wangq@zjzay.com (Q.W.); caoh@zjzay.com (H.C.); 2 College of Environment & Resource Science, Zhejiang University, Hangzhou 310058, China

**Keywords:** per- and polyfluoroalkyl substances, consumer products, migration, exposure assessment

## Abstract

Per- and polyfluoroalkyl substances (PFASs) have been widely used in the production of consumer products globally due to the excellent water and oil resistance and anti-fouling properties. The multiple toxic effects of some PFASs also pose a threat to human health and ecosystem, and the frequent use of certain consumer products increased the risk of human exposure to PFASs. More data on the occurrence, concentration, and migration of PFASs in consumer products is urgently needed to address the possible risks posed by exposure to consumer products. This paper reviews the PFAS concentrations found, the migration characteristics known, and the exposure risks of PFASs arising from several types of consumer products over the last five years. The types of consumer products considered here include food contact materials, textiles, and disposable personal hygiene products. The influence of different factors on the migration process of PFASs from these products are summarized and discussed. Additionally, the main approaches and models of exposure assessment are evaluated and summarized. Current challenges and future research prospects in this field are discussed with a view to providing guidance for the future assessment and regulation of PFASs in consumer products.

## 1. Introduction

PFASs refer to fluorinated substances containing at least one fully fluorinated methyl (-CF_3_) or methylene (-CF_2_-) group, excluding substances with hydrogen/chlorine/bromine/iodine attached to these carbon atoms according to Organization for Economic Co-operation and Development (OECD) [1]. There are thousands of PFAS molecules in a database of OECD [2]. The full names and abbreviations of the PFAS mentioned in this paper are summarized in Table 1. Due to the exceptional resistance to water, oil, and stains, PFASs have been widely applied in industrial applications, including antifouling and waterproof clothing, food contact materials (FCMs), firefighting foams, and additives in chemical manufacturing [3,4]. The strong C-F bond in their structure imparts PFASs with remarkable chemical stability, allowing them to persist in the environment for extended periods and migrate through various media [5,6]. Some PFAS precursors may occur involving the degradation of non-fluorine functional groups, including degradation processes such as oxidation, dealkylation, and defluorination, resulting in the formation of shorter chains of PFAA [7]. Over time, these hard-to-degrade PFASs accumulate in the environment and continue to enter the body through inhalation, diet, and other pathways. Numerous studies demonstrated that PFASs can be detected in the atmosphere, water, soil, and both human and animal bodies [8,9].

The harmful effects of some PFASs on human health and the environment raised widespread concern globally. The toxicity of PFASs varies significantly depending on their chemical structure. Long-chain PFASs (such as PFOA and PFOS) are widely recognized as having a higher risk of toxicity, while short-chain PFASs (such as PFBA) generally have lower environmental persistence and bioaccumulation, and their potential health risks still need further study [10,11]. Some PFASs exhibit the toxicities, including possible carcinogenicity (e.g., PFOA and PFOS) [12,13,14,15], immune system disorders (e.g., PFBA, PFNA, and PFOA) [16], organ toxicity (e.g., PFHxS) [17], and obesity (e.g., PFNA and PFOA) [18]. It is worth noting that there is still controversy about the carcinogenicity of PFAS [19]. Some in vitro studies have shown that PFASs can promote cancer by interfering with the endocrine system, inducing gene mutations, etc., but this mechanism has not been fully verified in population studies [20]. As our understanding of the toxicity of PFASs improved, many countries implemented controls on the use of these substances [21,22]. Following the prohibition of the production and use of traditional PFASs, such as PFOA and PFOS, a plenty of emerging PFASs are now being utilized as structurally modified alternatives [23,24,25]. However, studies indicated that these alternatives may also pose health risks to humans [3,6].

**Figure 1:**
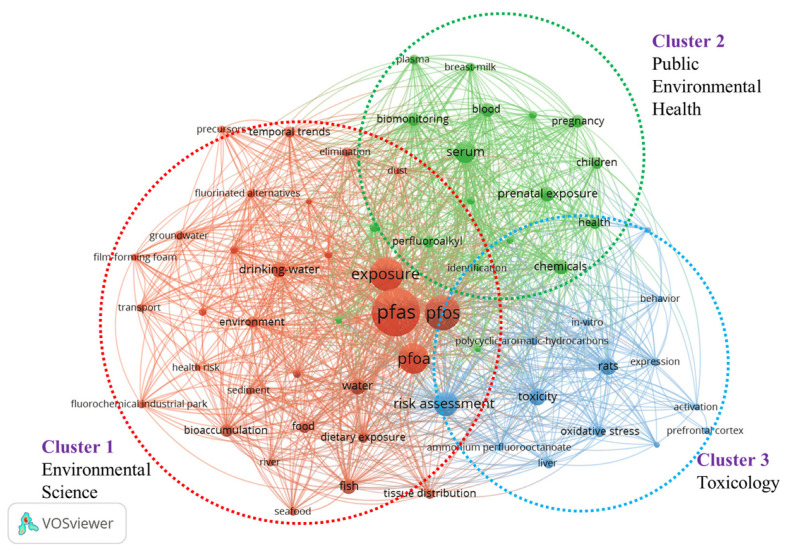
Keyword co-occurrence analysis among PFAS papers published in the last five years (data from Web of Science using “PFAS*”, “PFC”, and “exposure” as a keyword, data updated on 19 May 2024). The red, green, and blue circles represent clusters 1, 2, and 3, respectively.

**Figure 2:**
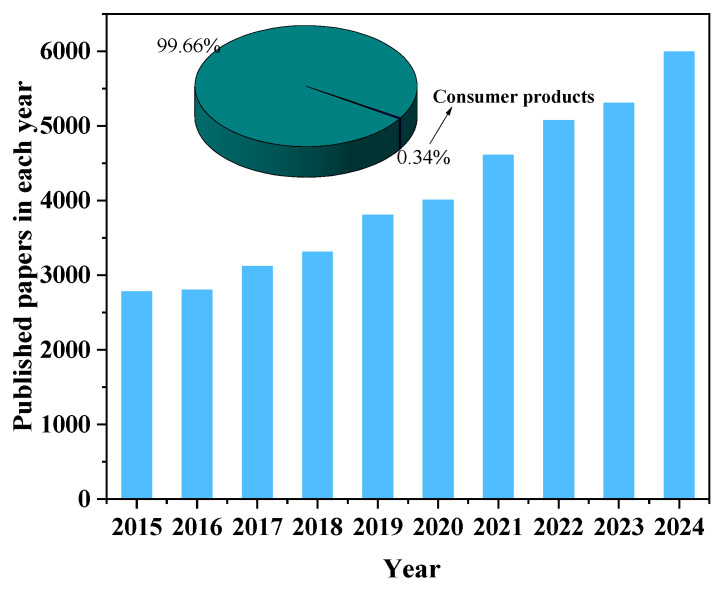
Paper numbers on PFASs published in the last five years (data from Web of Science using “PFAS*” and “PFC” as a keyword).

Humans are exposed to PFASs through various sources, including drinking water, food, and indoor environmental media. Consumer products also represent a potential route of PFAS exposure. To conduct a comprehensive analysis of the literature on PFAS exposure studies, we collected data (searched with “ PFAS*”, “PFC”, and “exposure”) in the database of the Web of Science, and 986 publications were recorded. VOS browser software (1.6.20) (https://www.vosviewer.com/, accessed on 10 May 2024) was applied, and a bibliometric map that graphically represents the co-occurrence network of keywords was created. As shown in Figure 1, the size of the dots in the image corresponds to the frequency with which the term appears. Three distinct clusters are identified, which mainly focus on the areas of environmental science, toxicology, and environmental public health. In recent years, the number of published papers on PFAS increased; however, research on PFAS in consumer products accounts for only 0.34% of the total literature (Figure 2). The distribution of PFAS in consumer products and the corresponding exposure risk are often overlooked. Previously, Matthias et al. [26] conducted a review analysis of PFAS in 115 consumer products in 2015. Mixtures of PFASs were found in varying concentrations across a wide range of consumer products, including FCMs, textiles, and leather samples, etc. It demonstrated the importance of screening and monitoring PFASs in consumer products. Dewapriya et al. [4] reviewed the types and concentrations of PFAS in household consumer products reported in the literature over the past decade, emphasizing that the presence of PFASs in consumer products was worthy of attention, but migration and risk assessment of PFASs in consumer products were still not fully explained. Therefore, there is currently limited knowledge about the occurrence of PFASs in consumer products, particularly with regard to possible migration of PFASs and subsequent human exposure.

This paper reviewed the detection, migration, and potential exposure risks of PFAS in common consumer items, including FCMs, textile products, and disposable sanitary products. The papers published between 2018 and 2024 in databases such as PubMed, Web of science, Google Scholar, and MDPI were surveyed. The key words are the combination of “PFAS” or “PFC” and “consumer goods”, “food packaging”, “food contact materials”, “textile”, “disposable product”, “exposure”, “migration”, etc. In order to facilitate data comparison and reliability, the quantitative studies were mainly screened based on liquid chromatography combined with mass spectrometry with appropriate QA/QC parameters. Additionally, this review introduced methods and models that are in line with current mainstream exposure risk studies. The paper also explores existing challenges and research trends in this field. The aim is to provide data support and a scientific basis for the management strategies of PFAS and the assurance of consumer product safety.

## 2. Detection of PFAS in Consumer Products

### 2.1. PFAS in FCMs

PFASs have been extensively utilized in FCMs due to their exceptional resistance to water and oil [27,28]. PFCA has been used as a surfactant in the polymerization of high-molecular-weight polymers such as PTFE, which had been employed in non-stick coatings for cookware since the 1960s [29]. Over a decade ago, concerns regarding the PFAS content in microwave popcorn packaging emerged, as these materials are subjected to high temperatures during use and may come into contact with fatty acids [30].

The detection details of PFASs in FCMs over the past five years are summarized in Table 2 [31,32,33,34,35,36,37,38]. The samples were collected from 2018 to 2023. In light of the recognized dangers of PFOA and PFOS [13], governments introduced policies to restrict their production and use. However, their presence continues to be reported in some studies. You et al. [31] studied FCMs sourced from marts and fast food restaurants of China. The highest concentrations of PFOS and PFOA detected in cartons and popcorn bags were 0.61 and 1.76 ng/cm^2^, respectively. Its presence is still a cause for concern despite the low levels. Due to the regulation of PFOS and PFOA, alternative substances such as GenX, 6:2 FTS, PFHxA, and PFBS emerged. Zabaleta et al. [32] collected paperboard FCMs and analyzed. PFHxA (2.1 ng/g) was detected in Spanish microwave popcorn bags, while six PFCAs were found in Chinese microwave popcorn bags, with concentrations ranging from 2.7 to 47 ng/g. Low concentrations of potential precursors (6:2 diPAP, 8:2 diPAP, 6:2 FTUCA, 8:2 FTUCA, and 7:3 FTCA) were detected in several greaseproof materials such as baking paper. Similar concentration levels were reported by Duenas-Mas et al. [33] (FCMs from French fast food restaurants, 0.010–3.3 ng/g), Boisacq et al. [34] (straws, Both <LOQ to 7.15 ng/g), and Timshina et al. [35] (straws, 0.043 ± 0.004 to 29.1 ± 1.66 ng/straw). The above results show that short-chain PFASs (C_4_–C_6_) wich, such as PFHxA, were detected more frequently with low concentration.

Meanwhile, some studies reported relatively high concentration levels in FCMs. For instance, Langberg et al. [36] collected FCMs manufactured in recycled paper from Norway, in which the total concentration of target PFASs detected ranged from 0.4 to 971 ng/g, dominated by SAmPAP diester and 6:2 FTS. Hoang et al. [37] conducted PFAS analysis on various FCMs collected from the Vietnamese market and found that a small number of cake paper tray samples had a high PFAS content (372–624 ng/g), and long-chain (C_8_–C_12_) PFCAs dominated. The total concentration of PFASs in popcorn bags collected by Wang et al. from China reached 377 ng/g, in which long-chain PFCAs (C_8_–C_18_) were significant [38]. The concentration of PFASs in the above study is several orders of magnitude higher than the previous survey results, which is related to sample number, sample representativeness, sampling area, etc.

### 2.2. PFAS in Textiles

PFASs have been widely used in textile-related applications since the 1950s, posing a significant source of exposure to PFASs [39]. Researchers from Non-Toxic Futures released a report that included an analysis of clothing, bedding, tablecloths, and napkins, revealing that PFAS was detected in 72% of anti-fouling and water-repellent products available on the market [40,41]. The PFASs in textiles of some studies are shown in Table 3 [42,43,44,45].

Zhu et al. [42] collected 160 textiles and identified 13 PFAAs. The concentration of PFAAs in flame-retardant textiles was the highest (59.4 ng/g), followed by waterproof textiles (12.9 ng/g) and baby clothing (2.33 ng/g). Among them, PFCA (C_4_–C_10_) accounted for at least three-quarters of the total PFAA content. Compared to conventional textiles, functional and outdoor textiles more frequently utilize PFAS as a surface-active agent. The maximum concentration of PFOA in carpet samples collected from a frequently used faculty office was 0.38 mg/kg [43]. Xia et al. [44] analyzed the levels of 49 PFASs in children’s textile products sold in stores across the United States and Canada. PFAS was detected in all products, with the highest concentrations of 6:2 FTOH. The total target concentration of PFAS in all products ranged from 0.250 to 153,000 ng/g. Notably, the total target PFAS content in school uniforms was significantly higher. Additionally, certain specialized occupations, such as firefighters, had a high frequency of exposure through clothing and personal protective equipment [45,46]. Peaslee et al. [45] tested PFASs in jackets and pants donated by fire departments in the United States and Australia. High fluorine levels were detected in all three linings tested. Textiles were in frequent contact with human skin, and combined with prolonged exposure to PFAS-treated textiles, can lead to skin absorption, posing a potential threat to human health [47,48]. Therefore, it is essential to clarify all ingredients in household items and clothing and to phase out the use of PFASs.

In recent years, there has been increasing global attention on PFAS precursors [45]. PFAS precursors are compounds that can be converted into PFASs through environmental degradation or biological metabolism, and under environmental conditions or biological metabolism, PFAS precursors may be converted into more toxic PFASs [49,50]. Zhu et al. found that textile extracts analyzed after oxidative treatment exhibited PFAA concentrations that were ten times higher than those measured before treatment [42]. Rewerts et al. [51] identified C_2_–C_7_ homologs of N-MeFASE and N-EtFASE by non-targeted analysis, which constituted 14–18% of the total perfluoroalkanes in the three textiles. Liagkouridis et al. [52] detected unknown side-chain fluorinated polymer (SFP) coatings in six medical textiles sourced from Sweden. Four products contained SFP based on C_6_-difluoride telomers, one contained C_4_-sulfonamide-based SFP, and another contained a C_8_-difluorotelomer. Therefore, given the extensive production capacity of PFAS precursors and their potential to generate persistent PFAA, neglecting the precursors of PFAS could lead to an underestimation of human and environmental exposure to PFAS. However, the wide variety of PFAS precursors and the lack of standard detection methods make it difficult to accurately determine their levels. At the same time, the migration and transformation mechanism of PFAS precursors in textiles is not fully understood, and it is difficult to assess their environmental behavior and health risks.

### 2.3. PFAS in Personal Hygiene Products

Personal disposable hygiene products (such as facemasks, sanitary napkins, diapers, and dental products, etc.) are a category of daily necessities with higher consumption frequency and longer use time. They are usually in direct contact with the body, which may facilitate the absorption of PFASs. Some findings of PFAS content in personal hygiene products were summarized in Table 4 [53,54,55,56].

PFASs may be used in the waterproof treatment of sanitary napkins or as an additive in the manufacturing process.Huang’s group [53] identified the use of personal hygiene products as a novel route for human exposure to PFAS. In total, 21 PFASs were detected on sanitary napkins (5.46 ng/g), pads (0.39 ng/g), tampons (<LOQ), diapers (4.72 ng/g), menstrual cups (14.95 ng/g), and germicides (0.16 ng/g). PFASs were detected in all diapers and menstrual cups. It is worth noting that the estimated exposure doses of PFOAs absorbed through the skin by infants (adults) with menstrual cups and diapers are 0.77 and 2.1 (1.2) ng/kg-bw/day, respectively, which is more than the normal ingestion of dust. To date, evaluations of PFAS in dental products have been limited to dental floss and tape. Massarsky et al. [54] estimated exposure to PFAS in convenience samples of leave-in dental products (such as night jackets and whitening trays) that remain in contact with the mouth for extended periods. The concentrations of PFBA and PFSA were found to be 0.67–0.83 ng/g and1.2–2.3 ng/g, respectively. PFASs also have been utilized in facial masks and certain anti-fogging products to minimize the condensation of water vapor. Muensterman et al. [55] collected nine masks for total fluorine and PFAS analysis, revealing that the total concentration of PFAS ranged from 15 to 2900 μg/m^2^. In multilayer masks, the outer layer exhibited the highest total fluorine content. Herkert et al. [56] characterized PFASs in anti-fogging sprays (*n* = 4), anti-fogging cloth (*n* = 5), and commercial fluorosurfactant formulations that are suspected to be used in the preparation of anti-fogging products (*n* = 2). FTOH and FTEO were detected in all products. The concentrations of PFAS in anti-fogging sprays and cloth reached up to 25,000 μg/mL and 185,000 μg/g, respectively. Total organic fluorine measurements for anti-fogging products ranged from 190 to 20,700 μg/mL in sprays and from 44,200 to 131,500 μg/g in cloth.

Although the PFAS content in sanitary napkins and dental products is low, the human exposure should not be ignored. Higher concentration levels in products such as masks, anti-fogging sprays, and cloth are of particular concern because they do not come into direct contact with human skin but may lead to additional inhalation exposure.

### 2.4. Brief Summary

According to the survey of the literature, there is lower PFAS content in FCMs and disposable sanitary products, while levels in textiles and spray products were higher. These findings identify a potential source of PFAS exposure for a wide range of consumers and provide a theoretical basis for manufacturing decisions regarding these products. The detection of PFAS in consumer products is currently mainly based in liquid chromatography-tandem mass spectrometry (LC-MS/MS), the emerging high-resolution mass spectrometry (HRMS), and corrected by isotope internal standard. These methods are highly sensitive with low LOD and LOQ. However, the expensive equipment, complex operation, and the detection of PFAS in complex substrates remains challenging. New mass spectrometry technology and sensors will be the future development direction. With advancements in technology, testing methods for fluorine species in products have also been continuously improved. For instance, Roesch et al. [57] combined micro-X-ray fluorescence (m-XRF) mapping with fluorine K-edge micro-X-ray absorption near-edge structure (m-XANES) spectroscopy to analyze the fluorine content of several consumer products. Tokranov et al. [43] proposed a new method for determining the percentage of fluorine atoms in a 0.01 μm layer on the surface of consumer products using X-ray photoelectron spectroscopy (XPS). The PFAS released in methanol extracts, quantified using conventional LC-MS/MS, represented only 1% of the fluorine detected in consumer products by XPS. This suggests that additional perfluorinated substances may be identified through spectroscopic methods. Therefore, further research is needed to fully understand the health risks associated with the use of these PFAS-containing products.

## 3. Migration of PFAS in Consumer Products

### 3.1. Migration of PFAS in FCMs

PFAS in FCMs can potentially transfer to food through a migration process, leading to direct dietary intake. However, risk assessment procedures primarily focused on the final concentration of PFAS in FCMs, without adequately distinguishing the contribution of migration. Identifying these potential pathways of exposure is critical for mitigating health risks [58]. Migration is influenced by several factors, including migration conditions (such as medium, temperature, and exposure time), food properties (such as pH and ionic flavoring, etc.), and type and characteristics of PFAS (including chain length, molecular weight, and concentration). Therefore, it is essential to evaluate the migration of PFASs from FCMs to food under various conditions. The experimental conditions and migrations for certain FCMs are summarized in Table 5 [32,35,59,60,61,62,63,64,65,66,67,68].

(1) Influence of migration condition

Medium. Most studies use chemical solvents as food simulants (FS) for migration tests [69]. Li et al. [59] investigated 36 PFASs that migrated from FCMs into 3% acetic acid, 10% ethanol, 50% ethanol, and olive oil, respectively. Some specific PFASs (0.01–0.05 mg/kg) were found in the migrants from approximately 5% of paper samples. Multilayer cardboard and coated cardboard were the main types of packaging in which PFASs were identified, with an average concentration of 0.02 mg/kg. Choi et al. [60] used 50% ethanol, 4% acetic acid, water, and n-heptane as simulants to assess the migration concentrations of frying pans, baking sheets, and baking paper. PFAS was not detected in the migration solutions of baking sheets, pots, rice cookers, and non-stick baking paper. A total of 7 PFASs were detected in the migration fluid of frying pans, with concentrations of PFNA measuring 1.76 μg/L and 2.12 μg/L, respectively. The testing frequency for the 50% ethanol simulant was the highest (2.56%), followed by 4% acetic acid (1.92%), water (0.96%), and n-heptane (0.96%). These findings suggest that alcoholic beverages or high-fat foods are most likely to facilitate the migration of PFAS from fluorocarbon resins in utensils.

In addition to using chemical solvents as FS, soli simulators that do not contain moisture or oil have also been employed to test the migration of PFAS in FCMs. Vera et al. [61] utilized Tenax^®^ as an FS to detect 11 PFAS in packaged samples, with migration concentrations ranging from 3.2 to 22.3 ng/g. However, there are notable differences between FS and actual food substrates, highlighting the urgent need for migration research based on real food substrates. Lerch et al. [62] investigated the effects of PFCA, PFSA, PAP, and FTOH in six FCMs on the migration into FS (50% and 20% ethanol) and actual foods (oatmeal, muffins, and tomato soup). The amount of PFCA and FTOH that migrated into 50% ethanol was significantly higher than the amount that migrated into real food, while FTOH did not migrate into 20% ethanol. Zabaleta et al. [32] also found that the amount of PFAS migrating into food, as determined using Tenax^®^, has been significantly underestimated, particularly for short-chain PFAS. Therefore, differences between FS-based migration tests and real food substrates may lead to inaccurate risk assessments.

Temperature. FMCs are consistently exposed to various high-temperature conditions. Timshina et al. [35] conducted a leaching experiment on straw with the highest levels of PFAS by immersing it in water at initial temperatures of 4, 20, and 90 °C, respectively. The results indicate no statistically significant differences among the various temperatures, as PFAS was not readily leached into the water. Toptanci et al. [63] analyzed the migration of PFAS and PFAA in 35 cookware samples sold in Turkey. The release of PFAS from non-stick cookware was investigated by examining the amount of simulation, temperature, and number of reuses. The migration rates of PFOA and PFOS in non-stick cookware were measured using 3% acetic acid simulators in different volumes (200, 500, and 1000 mL) at 70 °C for 2 h. PFOA was detected in six samples, with concentrations ranging from 2.12 to 8.86 ng/g. The migration ability of PFAS and PFAA increased with rising temperatures.

Ionic flavoring agents. Salt was an important addition to food and the cooking process. Consequently, understanding the impact of salt content on the migration of PFAS is of particular importance. In 2012, Chiang et al. [64] studied the migration of PFOA from non-stick pans at 125 °C and oil-resistant paper at 100 °C under high-temperature conditions typical of Chinese cooking, which included edible oils and ionic condiments such as salt, sauce, vinegar, and ketchup. The findings reveal that the use of ionic flavoring agents and oil in Chinese cooking increased PFOA migration by up to 1.2 ng/dm^2^ in cooking pots and 9.2 ng/dm^2^ in paper products compared to the use of oil alone. More recently, Mohamed et al. [65] compared the transfer of PFOS and PFOA from non-stick tableware to savory and non-savory foods, finding that the migration of foods with added salt was greater than that of foods without added salt.

Acidity and alkalinity. The acidity and alkalinity of food also influence the migration of PFAS. Mohamed et al. [65] investigated the migration of PFOS and PFOA from Teflon-coated containers to acidic foods, such as tomato sauce, and alkaline foods, such as white beans. The results indicate that the migration levels of PFOS (18.30 ug/kg) and PFOA (16.55 ug/kg) in tomato paste were higher compared to those in white beans (18.08 and 16.03 ug/kg), which were lower, respectively. PFASs exhibit greater mobility in acidic foods than in alkaline food substrates.

(2) Influence of PFAS properties.

Some studies demonstrated that the type and length of the fluorination chain significantly influence the migration of PFAS. For instance, Li et al. [39] identified that the main types of PFAS present in FCMs were straight-chain perfluoroacids and perfluoroalcohols. Timshina et al. [35] observed that short-chain PFASs, including PFBA (C_4_), PFPeA (C_5_), and PFHxA (C_6_), were nearly completely leachable in methanol migration experiments conducted with paper straws. In contrast, certain long-chain PFASs, such as PFTeDA (C_14_) and PFODA (C_18_), did not leach under these conditions. Whitehead et al. [66] performed a leaching experiment using a fluorinated container. Across all experiments, short-chain PFCAs exhibited the highest DF and concentration, while the analyzed concentration decreased as chain length increased. Zabaleta et al. [32] reported varying migration patterns based on carbon chain length: short-chain PFASs tended to migrate into 50% ethanol, whereas long-chain PFAS was more likely to migrate into 95% ethanol. These findings suggest that both increased chain length and food fat content contribute to the migration of PFAS from packaging materials into the food matrix.

Migration testing needs to simulate the conditions under which FCMs will be used in practice, including temperature, time, and food type, etc. Based on existing research, IT can be found that alcohol, high fat, acidic foods, and temperature increases may promote the migration of PFASs from FCMs. The carbon chain lengths showed different migration trends for different media. The relationship between PFAS functional groups and migration rule has not been clarified. It is important to clarify that there is a difference in the amount of migration between food simulants and real food substrates, so adequate consideration should be given when designing migration conditions. In addition, different countries and regions may differ in the choice of food simulators; for example, China defines food with a pH less than 5 as acidic food, and uses 4% acetic acid as the simulator. The European Union defines foods with a pH of less than 4.5 as acidic foods, using 3% acetic acid as a simulator [70,71]. The pH range of real foods may be wider; the composition of real foods is more complex than that of simulants and may contain a variety of organic and inorganic components that may have complex interactions with FCMs that affect the migration of PFAS. Detailed explanations and confirmatory experiments would provide valuable insights into the parameters that affect the migration of PFAS into food, thereby aiding in risk management. However, the complexity of actual use conditions makes the laboratory simulation have some uncertainty, and the migration patterns of PFAS into different types of food still require further clarification.

### 3.2. Migration of PFAS in Textiles

**Figure 3:**
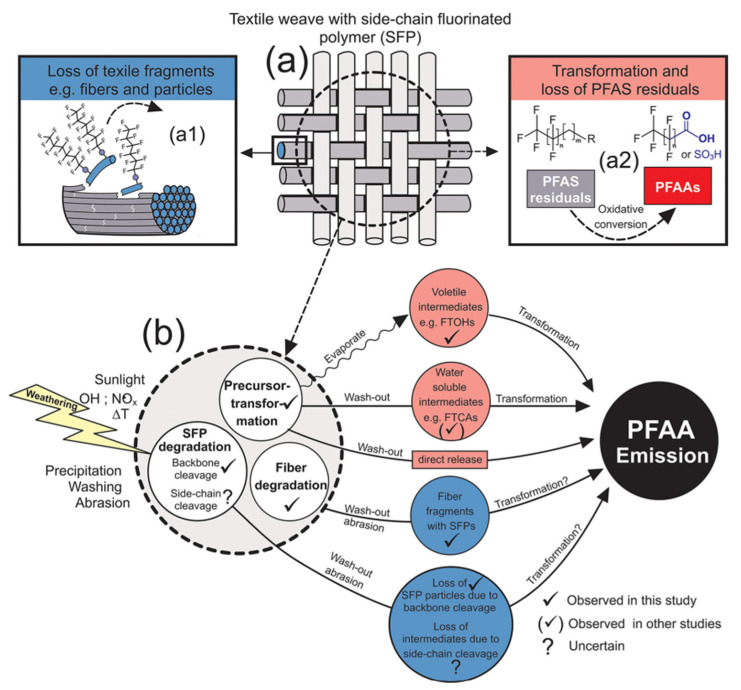
Schematic representation of loss mechanisms that that are likely to occur to (**a**) textiles with SFP finishes during weathering: (**a1**) Loss of larger textile fragments such as fibers and particles and (**a2**) the oxidative conversion of PFAS impurities. (**b**) Displays further a simplified summary of emission pathways that lead to emission and accumulation of PFAA in the environment [73].

Textiles are primarily in contact with the skin and possess properties that allow for long-term use and reusability. Peaslee et al. [45] identified and quantified individual PFAS in both new and used firefighting equipment. The analysis revealed that used gear exhibited lower levels of PFAS. Additionally, dust samples collected from textile storage areas indicated a direct loss of PFAS from fluoropolymers present in textiles. These findings suggest that PFAS in textiles can migrate during use, potentially exposing consumers to these substances. Research on PFAS migration in textiles predominantly concentrated on its transfer into the environment. During the use of outdoor clothing, PFAS can be released into the environment from durable water repellent (DWR) coatings. Veen et al. [72] evaluated the effects of aging, washing, and tumble drying on the extractable concentrations of PFAS in DWR-treated fabrics. The results indicate that the aging of coated fabrics led to an increase in the concentration and formation of PFAA, while washing resulted in a decrease in PFAA concentrations. Schellenberger et al. [73] conducted an outdoor weathering test on functional textiles in Sydney, investigating the emission of PFAS. The samples treated with water-resistant materials and SFP were exposed on a rooftop to various natural stressors, including direct sunlight, precipitation, wind, and heat for 6 months. The study examined the loss of PFAS-containing textile fragments during weathering, as well as the formation and loss of the low-molecular-weight PFAS. Potential emission pathways of PFAS were identified (Figure 3): (i) loss of larger fabric fragments, such as fibers and particles; (ii) degradation of SFP in textile fibers due to main-chain cleavage; (iii) oxidative transformation and cleavage of the fluorinated side chain of SFP, leading to the loss of low-molecular-weight PFAS; and (iv) oxidative conversion and impurity loss of mobile low-molecular-weight PFAS. These processes may occur simultaneously and are not easily distinguishable from one another. During weathering, due to the deterioration and loss of textile fibers and the degradation of SFP, most PFASs were lost in the form of SFP polymers, ultimately resulting in a loss of water resistance and color.

The widespread use of PFAS in textiles raised concerns about the potential risk of their migration to human skin. Studies have shown that PFAS can migrate from textiles to the surface of the skin through direct contact, dissolution of sweat, and friction, and may be absorbed into the body through the skin [74]. Infants and children, whose skin barrier function is not fully developed, are more sensitive to the risk of skin absorption of PFAS [75]. In particular, prolonged wearing of PFAS-containing clothing (e.g., outdoor sports wear, school uniforms) may increase cumulative exposure to PFAS. The skin absorption efficiency of PFAS is affected by its chemical structure, skin condition, and environmental conditions. For example, short-chain PFASs are more easily absorbed through the skin than long-chain PFASs (such as PFOA and PFOS) because of their smaller molecular weight and higher water solubility [76]. In addition, the presence of sweat may also promote the release of PFAS from textiles and enhance their skin permeability, especially under high-temperature or exercise conditions [77]. The risk of PFAS migration from textiles to human skin cannot be ignored, especially in certain populations and environments. Future studies are needed to further quantify the skin absorption efficiency of PFASs and their health effects, while strengthening the regulation of PFAS in textiles and the development of alternatives to reduce the risk of human exposure.

## 4. Exposure Risk Assessment

Exposure assessment is an important part of the risk assessment of chemical substances, which aims to quantify the dose of exposure of an individual or group to a certain chemical substance in a specific environment [78]. Exposure assessment can be carried out through biomonitoring or exposure model prediction.

### 4.1. Biological Monitoring

Biological monitoring refers to the direct detection of pollutant concentrations in human biological samples such as skin, blood, saliva, hair, nails, and others, in order to evaluate their levels within the human body. Human is exposed to PFAS through dietary intake, skin contact, and respiration, and PFASs in humans are involved in metabolic processes such as biotransformation and excretion [79]. Due to the stability of PFASs and their reabsorption properties in the body, many PFASs have low clearance rates, leading to their long-term accumulation in the body [80]. The half-life is related to the structure of PFASs, shorter chains of PFASs generally have shorter half-lives, while longer chains of PFASs are more difficult to remove [81]. In addition, functional groups also have an impact on the half-life and clearance of PFASs. For example, PFASs containing sulfonic acid groups (such as PFOS) usually have a longer half-life, and the strong hydrophilic and negatively charged properties of sulfonic acid groups give them a strong ability to bind to proteins (such as serum albumin) in the body, thus extending residence time in the body [82]. Statistical analyses of the data can reveal correlations between PFAS concentrations and specific diseases. Samples can be categorized into invasive and non-invasive types based on the methods used for sample collection.

#### 4.1.1. Invasive Samples

Blood. Due to the proteophilic properties of PFAS, blood was usually a good substrate for determining the exposure dose of PFAS [83,84,85,86]. Researchers can assess the relationship between the use of a product and the chemical by linking the concentration of PFAS in the serum of a population to the use of a product [87,88]. For example, to explore whether the use of CL increases exposure to PFAS in the general population, Kang et al. [89] analyzed data from 1660 adults aged 20 to 39 in the United States. The survey revealed that covariation-adjusted serum concentrations of PFOA and PFHxS in CL users (3.68 and 1.58 ng/mL, respectively) were significantly higher than those in non-users (3.27 and 1.30 ng/mL, respectively). Susmann et al. [88] selected representative samples from the United States to investigate the association between the consumption of fast food, restaurant food, homemade food, microwave popcorn, and serum levels of several PFAS. The results indicate that the caloric intake from foods consumed at home over the previous 24 h was significantly negatively associated with serum levels of all five PFAS. In contrast, meal consumption from fast food establishments and restaurants was linked to higher serum concentrations of PFAS. Additionally, based on 24 h and 12-month recall data, popcorn consumption was associated with significantly elevated serum levels of PFOA, PFNA, PFDA, and PFOS, with a 63% increase in PFDA among individuals who consumed popcorn daily over the past 12 months. The negative association between serum PFAS levels and foods consumed at home is attributed to reduced exposure to FCMs.

Target organs. Due to ethical limitations, there are very few reports on PFAS in human organs, and only a limited number of studies demonstrated that organs such as the liver and kidneys are prone to PFAS accumulation [90,91,92,93]. Mamsen et al. [94] collected blood, placental tissue, and fetal organs from 39 Danish women following pregnancy termination to assess their PFAS content. The substances detected in fetal organs included PFOS (0.6 ng/g), PFOA (0.2 ng/g), PFNA (0.1 ng/g), PFUnDa (0.1 ng/g), and PFDA (0.1 ng/g). A significant positive association was observed between exposure duration and the feto-maternal ratio for all five PFASs. The potential health hazards posed by these substances to the developing fetus remain unknown. Mamsen et al. [95] collected 225 fetal organs, including the heart, liver, lungs, central nervous system, and adipose tissue, from 78 embryos and fetuses ranging from 7 to 42 gestational weeks. In maternal serum samples, the highest concentration of PFOS was detected, and similarly, the highest concentrations of PFOS were found in embryonic or fetal tissues. PFHxS was detected in only a small number of fetuses. Furthermore, the ratio of PFOS, PFOA, and PFNA in the placenta to that in maternal serum increased throughout pregnancy, indicating bioaccumulation in the placenta. Grandjean’s research group [90] obtained and extracted tissue samples from forensic autopsies of 19 healthy adult subjects who experienced sudden death without elevated PFAS exposure, investigating the concentrations of PFAS in the liver, kidneys, lungs, spleen, brain, and whole blood of major toxicological target organs. There were minor differences in PFAS concentrations between the renal cortex and medulla, as well as between lung lobes. Organ concentrations of PFOS and PFNA correlated well with blood concentrations, while PFOA and PFHxS exhibited stronger correlations. Similarly, concentrations in the liver were closely related to those in other organs. However, the samples in studies examining the occurrence of PFAS in human organs were inadequate, and the findings may have been influenced by a lack of homogeneity.

#### 4.1.2. Non-Invasive Samples

Due to the invasive nature of blood collection, many study participants declined to donate blood samples, which often compromised the statistical significance of the limited samples available. Non-invasive techniques could reduce sample collection costs and discomfort for study subjects, while also facilitating the collection of samples. This shift made non-invasive substrates such as hair, nails, and urine more suitable for biological monitoring.

Human urine. Urine has been identified as a suitable biological sample for detection of those PFASs that are rapidly cleared from the human body [96,97]. The relationship between serum and urine concentrations of PFASs and airborne dust concentrations has been extensively studied. Zheng et al. [98] collected blood and urine samples from residents of Indiana and across the United States, measuring 47 PFAAs and their precursors. Ultra-short-chain (C_2_–C_3_) and short-chain (C_4_–C_7_) PFAAs exhibited the highest concentrations, accounting for 69–100% of the total PFAA concentration on average. Specifically, TFA (C_2_) and PFPrA (C_3_) were the main PFAA in most samples. This study demonstrated that ultra-short-chain and short-chain PFAAs are now prevalent in indoor environments and in humans, highlighting the need for further research on the potential adverse health effects of these exposures. Occupational exposure to PFASs emerged as a public health concern in recent years [99]. Peng et al. [100] collected serum and urine samples from 163 workers at five waste recycling plants, analyzing the concentrations of 21 PFASs. The average concentrations of these PFASs in urine and serum samples were 66.6 and 31.3 ng/mL, respectively. The concentrations of PFCA in urine were significantly higher than PFSA, especially for short-chain PFCA. Additionally, serum concentrations of PFAS in males were significantly higher than those in females, and seniority was positively correlated with serum concentrations of most PFASs. Urine can indicate exposure levels in the body without the direct transfer of external exposure agents. However, studies found that the concentrations of PFASs detected in urine are lower than those found in individuals with community or occupational exposure to contaminated sites [96]. The suitability of urine as a substrate for biomonitoring varies due to the efficiency of renal excretion and the individual’s living environment.

**Figure 4:**
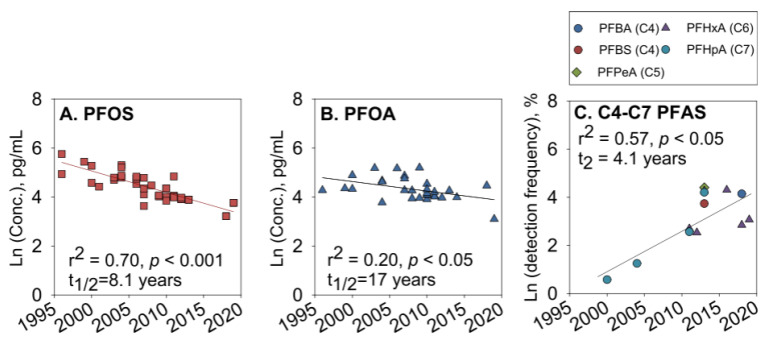
Changes in PFOS (**A**) and PFOA (**B**) concentrations (ln-transformed; pg·mL^−1^) and detection frequencies (normalized to the highest detection limit for each compound across all studies and ln-transformed, %) of short-chain PFAS (**C**) in breast milk during 1996–2019 [104].

Human milk. PFASs are transferred from maternal blood to breast milk, exposing infants to health risks through breastfeeding [101,102,103,104,105]. Criswell et al. [103] characterized the concentrations of 10 PFASs in the breast milk of a group of women from the New Hampshire Birth Cohort Study and compared the milk and plasma concentrations of 9 PFASs. The results indicate that approximately 6.5% of infants were exposed to PFAS through breast milk. Zheng et al. [104] analyzed the concentration levels of PFAS in the breast milk of American mothers and detected 16 different PFASs. The total target concentrations of PFAS in breast milk ranged from 52.0 to 1850 pg/mL, with significant levels of PFOS and PFOA. Two short-chain PFASs, PFHxA (9.69 pg/mL) and PFHpA (6.10 pg/mL), were detected in most samples. An analysis of available data on PFAS in breast milk from around the world between 1996 and 2019 showed that while the levels of PFOS and PFOA have been decreasing due to phasing out, the frequency of detection of currently used short-chain PFAS has been increasing (Figure 4). Limited information is available regarding how PFAS concentrations in breast milk change during lactation, despite their significance for cumulative infant exposure during breastfeeding. Blomberg et al. [106] measured PFAS levels in colostrum and mature milk samples from 77 women in Lonnebi and estimated monthly changes in concentrations over the first eight months of lactation. The concentration of PFAS varied between colostrum and mature milk. In women with low colostrum concentrations, PFAS levels tended to increase, while they remained constant or decreased in those with higher milk concentrations. Therefore, when modeling cumulative exposure to infants through breastfeeding, it is essential to consider differences in maternal exposure levels and changes in PFAS concentrations. Further research is needed to assess PFAS exposure from breastfeeding. Additionally, the composition of breast milk varies significantly between and within individuals and is influenced by numerous factors, including body lipids [107], reproductive history, and geographic region [108,109].

Hair and nails. PFAS can enter hair through three primary routes: passive diffusion from the bloodstream, diffusion from sweat or sebum secretions into the hair shaft, and deposition from the external environment [110]. Consequently, hair serves as a valuable biological indicator due to its non-invasive collection methods, ease of storage, and transportation. It can also reflect the overall levels of long-term internal and external exposure to these substances. Piva et al. [111] conducted a study on 20 PFASs in the hair of 86 Italian subjects. Among the analyzed population, 66.4% exhibited quantifiable levels of one or more PFAS molecules. The average total PFAS content was 0.1457 ng/g, while PFOA and PFOS were detected frequently with average concentrations of 0.1402 and 0.1155 ng/g, respectively. PFBA (0.3760 ng/g), PFNA (0.12 ng/g), and PFDA (0.541 ng/g) were detected, while PFUnDA and PFHxS were detected below the LOQ. The analysis revealed variations in PFAS concentration levels in the hair of individuals from different geographic regions, suggesting that hair could be utilized as a diagnostic tool to assess PFAS exposure on a regional scale. Nevertheless, it is important to consider that the concentrations of compounds measured in hair and nail samples may result from direct contact with the surrounding environment, including air, dust, and consumer products [112].

Overall, biological monitoring for PFAS primarily focused on human blood and urine. Geographic variations in blood PFAS concentrations among the population in the study area may be associated with dietary intake, drinking water sources, dust exposure, and levels of industrialization. Additionally, PFAS levels in human urine exhibited spatial differences and were influenced by factors such as PFAS chain length and sex. Notably, PFAS concentrations in breast milk were generally much lower than those found in human blood, potentially due to reduced transfer rates of these chemicals from the mother’s bloodstream to breast milk. To date, research has been limited regarding the use of human hair or nails as promising non-invasive methods for PFAS biomonitoring. However, exposure assessments by biological monitoring present several challenges, including difficulties in determination, high costs, and unclear specificity.

### 4.2. Exposure Assessment Models

The exposure assessment model is the core tool of chemical health risk assessment, which is used to quantify the level of population exposure to harmful substances through environmental, dietary, occupational, and other ways.

Traditional exposure assessment models. A specific exposure scenario is typically established, and the exposure within that scenario is estimated using relevant data (e.g., substance concentration), a range of exposure factors (e.g., duration of exposure, frequency of exposure, and respiratory rate), and the application of exposure models. The primary routes of human exposure to chemical pollutants include dermal contact, oral intake, and inhalation [113,114,115]. The exposure dose of each route can be estimated using the following equation [116,117,118]:

(1) $$EDI_{\mathrm{der}mal}=\frac{Q\times t\times F}{BW}$$

(2) $$EDI_{oral}=\frac{DC\times FC}{BW}$$

(3) $$EDI_{inhalation}=\frac{C\times IR\times t}{BW}$$

where *EDI* refers to the estimated daily exposure to PFAS via dermal contact, oral intake, and inhalation (ng/kg/day); *Q* represents the total mass of PFAS present on the skin (ng); *t* denotes the duration of exposure (h/day); F indicates the skin absorption fraction (%) of PFAS; *BW* is the individual’s body weight (kg); *DC* refers to the daily consumption (ng/day) of a specific product or food item; *FC* represents the concentration of PFAS in the product (ng/g); *C* denotes the concentration of individual PFAS in the air (ng/m^3^); and *IR* is the inhalation rate (m^3^/day).

Novel exposure assessment tools. As technology evolved, more accurate tools utilizing computer and algorithmic simulations enhanced exposure and risk assessments [119]. Regulators in various countries developed some models based on local data, exposure scenarios, and policy needs [120,121,122,123,124,125,126,127,128,129,130,131,132,133,134,135]. For example, the U.S. Centers for Disease Control and Prevention (CDC) and the U.S. Agency for Toxic Substances and Disease Registry (ATSDR) developed the PFAS Exposure Assessment Technical Tools (PEATT) to assist state, local, tribal, and territorial health departments with their PFAS biosurveillance activities [120]. The U.S. Environmental Protection Agency (EPA) uses a wide range of exposure assessment models in the health risk assessment of chemicals and pollutants, covering multiple exposure pathways such as air (The Air Pollutants Exposure (APEX) and Stochastic Human Exposure and Dose Simulation (SHEDS) models) [121], drinking water (EPANET model) [122], and food (Dietary Exposure Evaluation Model (DEEM)) [123]. In occupational and consumer product exposure assessments, EPA is promoting the development of high-throughput exposure prediction models through the RISK21 framework and ExpoCast programs. For example, the Consumer Product Exposure and Uptake (CPEO) model is used to assess skin contact and inhalation exposure to chemicals in consumer products (e.g., detergents, cosmetics).

Other countries and regions are also actively developing and applying exposure assessment models. The European Union, through the European Food Safety Authority (EFSA), issued standardized guidelines promoting models such as the Monte Carlo Risk Assessment (MCRA), which focus on assessing the cumulative effects of dietary exposure [124]. When Japan and South Korea introduced the American model, they developed an exposure prediction tool combined with a high-resolution geographic information system for their diet and living environment [125]. In recent years, China gradually established a multi-media exposure model through the New Environmental Management Measures for Chemical Substances, especially integrating source analysis and population flow data in the assessment of regional air pollutants such as PM2.5 [126]. Muensterman et al. [55] employed ConsExpo, an online tool developed by the Danish National Institute for Public Health and the Environment, to model exposure routes for inhalation and accidental ingestion of PFAS in masks. The exposure and risk estimates were found to be higher for children than for adults, and increased physical activity significantly elevated inhalation exposure. These preliminary findings suggest that prolonged use of masks treated with high levels of PFAS may represent a substantial source of exposure and pose a health risk. Different individuals may be exposed to varying PFAS due to the heterogeneity of exposure sources and patterns, with PFAS exposure sources systematically differing across populations.

In addition, the EPA developed a physiological pharmacokinetics (PBPK) model to simulate the absorption, distribution, metabolism, and excretion of chemical substances in the human body [127]. Goeden et al. developed a transgenerational toxicokinetic model to evaluate the transmission of PFOA between mother and infant, including placental and breast milk transmission. The model also took into account factors such as the mother’s water intake rate, body weight, and the infant’s breast milk intake rate to simulate changes in the serum concentration of PFOA in infants under different exposure scenarios [128]. Moreover, the PBPK model has been widely used in the assessment of chemical substances in consumer products such as cosmetics, cleaners, and plastic products. For example, Sarangapani et al. developed a PBPK model for toluene to assess its risk in occupational and consumer exposures [129]. Husøy et al. used PBPK models for cumulative exposure to PFO and blood concentrations in diet and personal care products [130]. In addition, chemicals in consumer products may be exposed simultaneously through multiple pathways (e.g., skin contact, inhalation, and ingestion). The PBPK model integrates multiple exposure data to provide a more comprehensive risk assessment. For example, Shatkin et al. used the PBPK model to assess multi-pathway exposure of nanomaterials in consumer products and their potential health risks [131]. Further, PBPK models are able to simulate exposure differences in different physiological states, such as children’s higher sensitivity to certain chemicals. Mielke et al. developed A child-specific PBPK model to assess the risk of exposure to bisphenol A in consumer products [132]. In the future, the development of PBPK models will benefit from high-throughput screening techniques, machine learning algorithms, and big data integration. For example, Russo et al. proposed to combine a PBPK model with an exposure prediction database to improve model applicability and prediction accuracy [133].

Common challenges to current models include data gaps (such as toxicological parameters for emerging pollutants), inadequate integration of exposure across media, and specific assessment needs of sensitive populations, such as children [134]. In recent years, the EPA actively explored the use of artificial intelligence and big data in exposure assessment. For example, the ExpoCast DB database integrates use patterns, physicochemical properties, and exposure data for chemical substances to support the development of machine learning models [134]. In the future, the convergence of artificial intelligence (such as machine learning-driven exposure prediction) and real-time monitoring technologies (such as wearable sensors) will increase model dynamics. In addition, there is a prominent need for international coordination, such as the GENERIC exposure assessment framework promoted by the OECD, which attempts to harmonize model input parameters across countries to reduce trade barriers.

### 4.3. Risk Characterization

The purpose of risk characterization is to synthesize the results of hazard identification, dose–response assessment, and exposure assessment to assess the potential risks of a chemical substance to human health. A variety of quantitative and qualitative methods are widely used at home and abroad for risk characterization. The hazard quotient (HQ) [135] and margin of exposure (MOE) [31] are two commonly used indicators. The HQ was defined as the ratio of the actual concentration of a pollutant to the predicted concentration at which no adverse effects are expected [118,136]. When HQ < 1, the risk is considered acceptable. When HQ is greater than or equal to 1, health risks may exist [137]. The MOE of a substance is defined as the ratio of its level of no observed adverse effects to the theoretical, predicted, or estimated dose or concentration resulting from human ingestion. A higher MOE indicates a lower risk. Generally, MOE > 100 is considered an acceptable risk [137]. The HQ method is simple and intuitive, and is suitable for non-carcinogenic risk assessment of a single chemical substance, but it does not consider the combined exposure effects of multiple chemical substances. The MOE approach has the advantage of reflecting uncertainty in the dose–response relationship, but it relies on high-quality toxicological data. In addition, most of the existing methods are based on a single exposure pathway, making it difficult to fully reflect the actual exposure scenario. Future studies need to incorporate multi-pathway exposure models, probabilistic risk assessment, and systematic toxicological approaches to improve the accuracy and reliability of risk characterization.

## 5. Limitations and Perspectives

This paper reviews the latest advancements in the evaluation of residues, migration, and human exposure of PFASs in consumer products. PFASs in consumer products have often been overlooked due to their low concentrations. However, with the advancement of research, multiple PFASs have been detected in FCMs, textiles, and disposable products. While some studies investigated PFAS migration in FCMs, the migration of PFASs in other consumer products remains unclear, hindering further exposure and risk assessments. Currently, research on the risks associated with PFAS in consumer products faces several challenges.

### 5.1. Accurate Detection of PFAS

Although numerous methods have been developed for detecting PFAS in the environment, the presence of PFAS in consumer products is characterized by low concentrations and complex matrices, resulting in many PFASs remaining largely unmonitored. The identification and quantification of PFAS in consumer products present significant challenges regarding accuracy, sensitivity, matrix effects, and practicality.

Limitations: (1) Low concentrations: PFASs in consumer products often exist at trace levels, requiring highly sensitive analytical techniques such as liquid chromatography-tandem mass spectrometry (LC-MS/MS) or high-resolution mass spectrometry (HRMS). However, even these advanced methods may struggle to detect ultra-trace levels of certain PFASs. (2) Complex matrices: Consumer products such as textiles, food packaging, and cosmetics contain complex chemical compositions that can interfere with PFAS detection. For example, dyes, additives, and other contaminants may mask PFAS signals or produce false positives. (3) Lack of standardized methods: There is currently no universally accepted standard method for extracting and detecting PFAS in consumer products. This lack of standardization leads to variability in results across studies, making it difficult to compare data and draw consistent conclusions.

Perspectives: (1) Development of advanced analytical techniques: research should focus on improving the sensitivity and selectivity of detection methods, such as developing new ionization techniques or enhancing sample preparation protocols to reduce matrix effects. (2) Establishment of standard protocols: International collaboration is needed to establish standardized extraction and detection methods for PFASs in various consumer products. This would enable real-time monitoring of PFAS contamination, accurate assessment of exposure risks, and effective investigation and control of pollution sources. (3) Expansion of monitoring programs: governments and regulatory agencies should expand monitoring programs to include a wider range of PFASs, particularly emerging and less-studied compounds, to better understand their prevalence and potential risks.

### 5.2. Applicable Evaluation Models

Quantifying an individual’s cumulative exposure burden to PFASs and their mixtures is crucial for health risk assessment outcomes. However, variations in model parameters arise from the heterogeneity of exposure sources and modes. The characteristics of the factors contributing to these differences in systemic exposure may be unknown, or they may result from a combination of these factors.

Limitations: (1) Heterogeneity of exposure sources: PFAS exposure can occur through multiple pathways, including ingestion (e.g., food and water), inhalation (e.g., dust and air), and dermal contact (e.g., textiles and cosmetics). Each pathway may involve different PFAS compounds and concentrations, complicating exposure assessments. (2) Lack of real-world data: Many existing exposure models rely on laboratory-based migration experiments, which may not accurately reflect real-world conditions. For example, factors such as temperature, humidity, and mechanical stress can influence PFAS migration in consumer products but are often not fully accounted for in experimental setups. (3) Cumulative exposure assessment: Most models focus on individual PFAS compounds, but humans are typically exposed to mixtures of PFASs. The interactions between different PFASs and their combined effects on human health are poorly understood.

Perspectives: (1) Real-world migration studies: More migration experiments based on real-world scenarios need to be conducted to obtain accurate migration parameters. For example, studies should simulate typical usage conditions for textiles, food packaging, and other consumer products to better understand how PFASs migrate under realistic conditions. (2) Development of mixture models: researchers should develop models that account for cumulative exposure to PFAS mixtures, incorporating data on the interactions between different compounds and their combined toxicological effects. (3) Integration of exposure pathways: Future models should integrate multiple exposure pathways to provide a more comprehensive assessment of PFAS exposure. This could involve combining data from environmental monitoring, biomonitoring, and consumer product testing.

### 5.3. Lack of Toxicity Data

The types and concentrations of PFAS in consumer products vary by country and manufacturer, and national legislation is often inconsistent and complex. More comprehensive toxicity data on PFAS, including information on impurities and contaminants, is essential for conducting accurate risk assessments.

Limitations: (1) Diverse PFAS compounds: There are thousands of PFAS compounds, each with potentially different toxicity profiles. However, toxicity data are primarily available for a few well-studied compounds, such as PFOS and PFOA, while data on emerging PFAS are scarce. (2) Inconsistent regulations: Different countries have varying regulations regarding PFASs in consumer products, leading to inconsistencies in safety thresholds and exposure limits. For example, some countries banned specific PFASs, while others continue to allow their use. (3) Limited data on mixtures: Most toxicity studies focus on individual PFAS compounds, but humans are exposed to mixtures of PFASs. The combined effects of these mixtures are poorly understood, making it difficult to assess their overall health risks.

Perspectives: (1) Expansion of toxicity studies: Research should prioritize toxicity studies on a wider range of PFASs, particularly emerging compounds and mixtures. This includes investigating both acute and chronic effects, as well as potential endocrine-disrupting properties. (2) Harmonization of regulations: International efforts are needed to harmonize regulations and establish consistent safety thresholds for PFASs in consumer products. This could involve collaboration between governments, industry, and academia to develop standardized risk assessment frameworks. (3) Development of alternative materials: Given the potential risks associated with PFASs, research should focus on developing and commercializing safer alternatives for use in consumer products. This includes exploring non-fluorinated materials that can provide similar performance without the associated health and environmental risks.

Overall, the evaluation of PFAS residues, migration, and human exposure in consumer products is a complex and evolving field. This review enhanced our understanding of the occurrence, migration, exposure pathways, and health risks associated with PFASs in consumer products. While significant progress has been made in detecting PFASs and understanding their migration patterns, several challenges remain. Future research needs to develop new detection techniques, migration experimental methods, and exposure assessment models to improve the accuracy and practicability of research, as well as establish a global PFAS database to share detection, migration and toxicity data, and facilitate scientific research and policy development.

## Figures and Tables

**Table 1:** List of analyte and abbreviations.

Analyte	Abbreviation	Analyte	Abbreviation
Perfluoroalkyl acid	PFAA	N-ethyl perfluoroalkane sulfinesulfaminoethanol	N-EtFASE
Perfluorooctanoic acid	PFOA	6:2 fluoromeric alcohol	6:2 FTOH
Perfluorooctyl sulfonate	PFOS	Fluoropolymer alcohol	FTOH
Perfluoroalkyl carboxylic acids	PFCA	Fluoropolymer ethoxylic acid	FTEO
Polytetrafluoroethylene	PTFE	Perfluorononanoic acid	PFNA
Hexafluoropropylene oxide-dimer acid	GenX	Perfluorovaleric acid	PFPeA
6:2 fluorotelomeric sulfonic acid	6:2 FTS	Perfluorotetradecanoic acid	PFTeDA
Perfluorohexyl sulfonate	PFHxS	Perfluorooctadecanoic acid	PFODA
Perfluorobutanesulfonic acid	PFBS	Perfluoroodecanoic acid	PFDA
Perfluorohexanoic acid	PFHxA	Perfluoroundecanoic acid	PFUnDa
6:2 fluorotelomer phosphate diester	6:2 diPAP	Perfluoropropionic acid	PFPrA
8:2 fluorotelomer phosphate diester	8:2 diPAP	Trifluoroacetic acid	TFA
2H-perfluoro-2-octenoic acid	6:2 FTUCA	Perfluoroheptanoic acid	PFHpA
Perfluorosulfonic acid	PFSA	Perfluorododecanoic acid	PFDoA
Polyfluoroalkyl phosphate	PAP	Perfluorotridecanoic acid	PFTrDA
Perfluoroobutyric acid	PFBA	2H-Perfluoro-2-decenoic acid	8:2 FTUCA
Perfluoroalkyl sulfonic acid	PFSA	3-perfluoroheptyl propanoic acid	7:3 FTCA
N-methyl perfluoroalkane sulfinyl aminoethanol	N-MeFASE		

**Table 2:** Summary of PFAS content in FCMs.

Samples	Analytes	Concentration	Region	Sample Collection Year	Analytical Methods	Ref.
Cartons and popcorn bags	PFOS PFOA	0.61 ng/cm^2^ 1.76 ng/cm^2^	China	2018–2019	/	[31]
Baking paper	6:2 diPAP	2.1 ± 0.5 ng/g	Spain	2018–2019	(1) Instrument: LC-MS/MS (2) Extraction: Ultrasound with methanol (1% acetic acid) and cleaned-up with ENVI-Carb cartridges (3) Internal standards: Yes (4) LOD: 0.7–3.5 ng/g (5) LOQ: /	[32]
Muffin cup	6:2 diPAP	7 ± 1 ng/g	Spain	2018–2019		[32]
Cardboard cup	6:2 diPAP	2.2 ± 0.2 ng/g	Spain	2018–2019		[32]
Burger wrapper	6:2 diPAP 8:2 diPAP	7 ± 2 ng/g 4 ± 1 ng/g	Spain	2018–2019		[32]
French fries wrapper	6:2 FTUCA	1.1 ± 0.1 ng/g	Spain	2018–2019		[32]
Microwave popcorn bags	PFHxA	2.1± 0.4 ng/g	Spain	2019		[32]
Microwave popcorn bags	PFPeA, PFHxA, PFHpA, PFOA, PFNA, PFDA,	2.7 ± 0.6 to 47 ± 10 ng/g	China	2019		[32]
Greaseproof materials	6:2 FTUCA 8:2 FTUCA 7:3 FTCA	3.1 ± 0.5 ng/g 12 ± 1 ng/g 1.2 ± 0.1 ng/g	China	2019		[32]
Fast food packaging	PFHxA 6:2 FTS 6:2/8:2 diPAP	0.042–3.3 ng/g 0.011–0.1 ng/g 0.010–1.9 ng/g	French	2021	(1) Instrument: LC-MS/MS (2) Extraction: Ultrasound with methanol and cleaned-up by ENVI-Carb cartridges (3) Internal standards: Yes (4) LOD: 0.0005–0.005 ng/g (5) LOQ: 0.002–0.02 ng/g	[33]
Paper-based straws	29 PFAS	<LOQ to 7.15 ng/g	Belgium	Not mentioned (paper published in 2023)	(1) Instrument: UPLC-MS/MS (2) Extraction: Ultrasound with methanol and cleaned-up by ENVI-Carb cartridges (3) Internal standards: Yes (4) LOD:/ (5) LOQ: 0.009–1.060 ng/g	[33]
Bamboo straws	29 PFAS	<LOQ to 3.47 ng/g	Belgium			[34]
Glass straws	29 PFAS	<LOQ to 6.65 ng/g	Belgium			[34]
Straws	21 PFAS	0.043 ± 0.004 to 29.1 ± 1.66 ng/straw	the United States Mexico China Vietnam and unknown	2019–2020	(1) Instrument: UHPLC-MS/MS (2) Extraction: Swirl with 0.3% methanolic ammonium hydroxide and centrifuge (3) Internal standards: Yes (4) LOD:/ (5) LOQ: /	[35]
Paper product (including FCMs)	37 PFAS	0.4–971 ng/g	Norway	2021	(1) Instrument: UPLC-QqQ-MS (2) Extraction: Ultrasound with ethyl acetate (3) Internal standards: Yes (4) LOD:/ (5) LOQ: 0.03 to 1.4 ng/g	[36]
Mochi paper tray	17 PFAS	372–624 ng/g	Vietnam	Not mentioned (paper published in 2023)	(1) Instrument: LC-MS/MS (2) Extraction: ultrasonicated with methanol at 60 °C for 2 h (3) Internal standards: Yes (4) LOD: 0.007 ng/g for PFBA (5) LOQ: /	[37]
Food contact paper and plastics	21 PFAS	3.2 to 377 ng/g	China	2022	(1) Instrument: HPLC-MS/MS (2) Extraction: ultrasonicated with methanol and cleaned-up by Oasis WAX cartridges (3) Internal standards: Yes (4) LOD: 0.1–5.8 ng/L (5) LOQ: 0.35–19.4 ng/L	[38]

/ means not mentioned.

**Table 3:** Summary of PFAS content in textiles.

Samples	Analytes	Concentration	Region	Sample Collection Year	Analytical Methods	Ref.
Flame retardant textiles	13 PFAA	59.4 ng/g	Albany, NY, USA	2016–2019	(1) Instrument: HPLC-MS/MS (2) Extraction: extracted twice with methanol and ethyl acetate by shaking at 180 strokes/min for 2 h; (3) Internal standards: Yes (4) LOD:/ (5) LOQ: 0.025–0.250 ng/g	[42]
Waterproof textiles	13 PFAA	12.9 ng/g				[42]
Baby clothing	13 PFAA	2.33 ng/g				[42]
Carpets	PFOA	0.38 mg/kg	the United States	Not mentioned (paper published in 2019)	(1) Instrument: LC-MS/MS (2) Extraction: extracted with methanol by shaking for 1 h and bath sonicated at 40 °C for 2 h; (3) Internal standards: Yes (4) LOD:/ (5) LOQ: 0.063–3.7 ng/g	[43]
School uniforms, waists, hats, stroller covers, and swimsuits	49 PFAS	0.250 to 153,000 ng/g	the United States and Canada	2020–2021	(1) Instrument: LC-MS/MS and GC-MS (2) Extraction: extracted with methanol by shaking for 1 h and bath sonicated at 40 °C for 2 h; (3) Internal standards: Yes (4) LOD:/ (5) LOQ: 0.01–3.49 ng/g	[44]
Jackets and pants of fireman	24 PFAS	2–850 ppb	the United States and Australia	2002–2017	(1) Instrument: LC-MS/MS (2) Extraction: sonicating overnight with NaOH solution and cleaned-up with WAX cartridges; (3) Internal standards: Yes (4) LOD:/ (5) LOQ: 1.12–13.2 ng/g	[45]

**Table 4:** Summary of PFAS content in personal hygiene products.

Samples	Analytes	Concentration	Region	Sample Collection Year	Analytical Methods	Ref.
Sanitary napkins Sanitary pads Tampons Diapers Menstrual cups Germicide	21 PFAS	5.46 ng/g 0.39 ng/g <LOQ ng/g 4.72 ng/g 14.95 ng/g 0.16 ng/g	China	2022	(1) Instrument: UHPLC-HRMS (2) Extraction: vortexed and ultrasonicated with 0.3% (*v*/*v*) ammonium hydroxide in methanol (3) Internal standards: Yes (4) LOD: 0.004–3.44 ng/g (5) LOQ: 0.01–9.47 ng/g	[53]
Night jackets and whitening trays	PFBA	0.67–0.83 ng/g	the United States	Not mentioned (paper published in 2024)	(1) Instrument: (2) Extraction: extracted with methanol (0.4% KOH) (3) Internal standards:/ (4) LOD:/ (5) LOQ: /	[54]
Night jackets and whitening trays	PFOS	1.2–2.3 ng/g	the United States			[54]
Masks	25 PFAS	15 to 2900 μg/m^2^	the United States	Not mentioned (paper published in 2022)	(1) Instrument: LC-qTOF (2) Extraction: shaked with methanol of 60–65 °C (3) Internal standards: Yes (4) LOD: 0.5–12 µg/m^2^ (5) LOQ: 1.7–4.2 µg/m^2^	[55]
Anti-fogging spray products	16 PFAS	up to 25,000 μg/mL	the United States	Not mentioned (paper published in 2022)	(1) Instrument: LC-MS/MS, GC-HRMS and HPLC-HRMS (2) Extraction: Sprays were extrac with acetone, Cloths were treated with HeX/DCM (3) Internal standards: Yes (4) LOD:/ (5) LOQ: /	[56]
Anti-fogging cloth products	16 PFAS	up to 185,000 μg/g				

**Table 5:** Summary of migration conditions and amounts of PFAS in FCMs.

Samples	Analytes	Migration Matrix	Amount of Migration	Temperature (°C)	Time	Ref.
Paper bag	9 PFAS	Tenax^®^	0.796 to 9.424 ng/g	60	10 days	[32]
			0.796 to 7.068 ng/g	150	15 min	
Straws	Total PFAS	Water	1.53 ± 0.122 ng/straw	4, 20, 90	/	[35]
Multilayer cardboard and coated cardboard	36 PFAS	3% acetic acid 10% ethanol 50% ethanol olive oil	10 to 50 ng/g	70	2 h	[59]
Frying pans	16 PFAS	Water or corn oil	0.728 to 3.052 µg/L	170~190	/	[60]
Baking utensils	16 PFAS	Water or corn oil	1.758 to 2.122 µg/L	170~190	/	[60]
Cardboard, biopolymer, paper, and Teflon trays	11 PFAS	Tenax^®^	3.2 to 22.3 ng/g	40	3 days	[61]
Paper plate	PFCAs/PFSAs, PAPs, FTOHs	Oatmeal Porridge	4.26 ± 1.24 ng/g	800 W microwave	1 V min	[62]
		Tomato Soup	11.3 ± 1.37 ng/g			
Muffin cup	PFCAs/PFSAs, PAPs, FTOHs	Muffin	3.41 ± 0.65 ng/g	200	13 min	[62]
Non-stick cookware	PFOA	3% acetic acid	2.12 to 8.86 ng/g	70	2 h	[63]
Coated cook pans	PFOA	edible oil and ionic condiments (salt, sauce, vinegar, and ketchup)	Up to1.2 ng/dm^2^	125 ± 5	15 min	[64]
Oil-resistant paper	PFOA		Up to 9.2 ng/dm^2^	100 ± 5	15 min	[64]
Teflon-coated containers	PFOS PFOA	Tomatoes pasta with salt	18.30 ng/g 16.55 ng/g	Cooking condition	1 time	[65]
Teflon-coated containers	PFOS PFOA	White dry beans	18.08 ng/g 16.03 ng/g	Cooking condition	1 time	[65]
Fluorine-containing containers	20 PFAS	Water Methanol Acetone	0.99 ± 0.46 ng/g 69.72 ± 7.75 ng/g 50.13 ± 4.41 ng/g	Room temperature	7 days	[66]
		Water	26.88 ± 4.21 ng/g	50	7 days	
		Olive oil Ketchup Mayonnaise	2.66 ± 0.82 ng/g 5.95 ± 1.59 ng/g 7.19 ± 3.39 ng/g	Room temperature	7 days	
		Olive oil Ketchup Mayonnaise	5.63 ± 0.42 ng/g 55.25 ± 11.87 ng/g 31.52 ± 4.62 ng/g	50	7 days	
Baking paper and aluminum foil	14 PFAS	Ethanol 95% acetic acid 3%	/ 1.43 to 13.87 ng/dm^2^	60	15 min	[67]
Paper and board	14 PFAS	Milli-Q Water and 95% Ethanol	0.001 μg/kg of PFOA up to 0.15 μg/kg of PFBA	20–80	30 min–24 h	[68]
Paper analogues	14 PFAS		0.003 μg/kg of PFPeA up to 0.29 μg/kg of PFBA			

## Data Availability

No new data were created or analyzed in this study.
